# Research on the ability of propionic acid and vitamin B12 biosynthesis by *Propionibacterium freudenreichii* strain T82

**DOI:** 10.1007/s10482-017-0991-7

**Published:** 2017-11-25

**Authors:** Kamil Piwowarek, Edyta Lipińska, Elżbieta Hać-Szymańczuk, Anna Bzducha-Wróbel, Alicja Synowiec

**Affiliations:** Department of Biotechnology, Microbiology and Food Evaluation, Division of Food Biotechnology and Microbiology, Faculty of Food Sciences, Warsaw University of Life Sciences – SGGW (WULS-SGGW), Nowoursynowska 159c Street, 02-776 Warsaw, Poland

**Keywords:** Propionic acid, Acetic acid, Vitamin B12, *Propionibacterium*, Carbon sources, DoE

## Abstract

The purpose of this study was to determine the potential for biosynthesis of propionic acid and vitamin B12 by *Propionibacterium freudenreichii* T82 in a medium containing various sources of carbon (glucose, fructose, and saccharose). These sugars are present in apple pomaces, which are the waste from the production of apple juice. Using statistical analysis design of experiments (DoE), the results allowed us to determine which sugars (carbon sources) exert the most beneficial influence on the biosynthesis of propionic acid and cobalamin. The highest production of propionic acid by the tested bacterial strain was obtained in a medium in which glucose accounted for at least 50% of the available carbon sources. Depending on the culture medium, the concentration of this metabolite ranged from 23 to 40 g/L. *P. freudenreichii* T82 produced the smallest amount of acid in medium in which the dominant nutrient source was saccharose. The results obtained indicated an inverse relationship between the amount of acid produced by the bacteria and vitamin B12 biosynthesis. Because of the high efficiency of propionic acid biosynthesis by *P. freudenreichii* T82, the prospect of using this strain to obtain propionate with the simultaneous disposal of waste materials (such as apple pomaces) which contain glucose and/or fructose is very promising.

## Introduction

Bacteria of genus *Propionibacterium* have been traditionally divided into two groups: skin (acnes) and classic (dairy). Classical strains include, among others, the species *Propionibacterium acidipropionici, Propionibacterium jensenii*, *Propionibacterium thoenii* and *Propionibacterium freudenreichii* (ssp. *shermanii*, ssp. *freudenreichii*) (Meile et al. 1999), of which the first three have recently been reclassified as members of the genus *Acidipropionibacterium* (Scholz and Kilian 2016). Classical *Propionibacterium* are a source of useful metabolites such as propionic acid and vitamin B12 (Meile et al. 1999; Patrick and McDowell 2015).

Propionic acid is used to inhibit the growth of yeast and molds in prepacked sliced bread, rye bread, breads with reduced calories, and partially baked rolls, pita bread, pastry products and animal feed. Propionic acid also is an essential indirect component in production process of the cellulose fibers, herbicides, perfumes, and pharmaceuticals (Suomalainen and Mäyrä-Makinen 1999, Gwiazdowski and Gwiazdowska 2008). Propionic acid for industrial purposes is currently synthesised only in chemical processes, as this is still more economical than microbial processes using propionic acid bacteria. However, due to the serious environmental damage that can be caused by chemical production of propionic acid, as well as due to the rise in demand for natural and ecological food products, there is an increasing demand for the microbial production of propionic acid, along with the desirability of using waste materials. This should reduce the cost of natural production of propionc acid and make it profitable and should have environmental benefits (Baumann and Westermann 2016). Such waste materials could include apple pomaces, which contain sugars (glucose, fructose, saccharose), proteins, pectins, fiber, vitamins and organic acids, which may affect the efficiency of synthesis of propionic acid or cobalamin by *Propionibacterium* spp. and relatives.

Bacteria of the genus *Propionibacterium* and relatives seem the most appropriate for the biotechnological production of propionic acid. Due to their wide variety of enzymatic systems, they can use carbon from various sources, for example: glucose (Himmi et al. 2000), xylose (Carrondo et al. 1988), lactose (Hsu and Yang 1991), saccharose (Quesada-Chanto et al. 1994), lactic acid (Barbirato et al. 1997), maltose (Zhu et al. 2012) and whey (Lewis and Yang 1992). These bacteria can be used in the reprocessing of waste materials including glycerol (Yazdani and Gonzales 2007; Zhu et al. 2010), hemicellulose hydrolysates (Ramsay et al. 1998), corn flour (Huang et al. 2002) and cane molasses (Feng et al. 2011).

The most favourable bacteria for industrial production of vitamin B12—due to their Generally Recognized as Safe status (GRAS) and ability to synthesise active forms of this metabolite—may be strains of *P. freudenreichii*. However, currently commercially produced cobalamin uses a genetically modified strain of *Pseudomonas denitrificans* (without GRAS status) (Blanche et al. 1989; Miyano et al. 2000; Roman et al. 2001). Because in many parts of the world the use of genetically modified organisms (GMOs) still causes controversies, the search for appropriate carbon source and microbes capable of producing this metabolite with high efficiency, without interfering with their genomes, remains a challenge.

In these studies we used the mixture design method of DoE (Design of Experiments) as a tool to facilitate optimising composition of carbon sources in the culture medium of *P. freudenreichii* (Mason et al. 1989). The statistical method of DoE provides an efficient plan for experimentation, so that many factors influencing a process can be simultaneously studied. Statistical approaches have become useful tools for understanding the interactions among various parameters with a minimum number of experiments (Gupta et al. 2007). The DoE method has many benefits in comparison to conventional one-factor-at-a-time methods, which can fail to locate optimum parameters, because it is impossible to describe a possible effect of interactions between factors (Gupta et al. 2004; Teng and Xu 2008; Fabiszewska et al. 2015). The DoE method has been applied to optimise the synthesis of lipase by *Yarrowia lipolitica* (Fabiszewska et al. 2015).

The aim of this study was to determine the potential for biosynthesis of propionic acid and vitamin B12 by *P. freudenreichii* strain T82 and apply an unconventional statistical approach to develop the optimal composition of sugars in the medium which might stimulate high production of metabolites by this strain. The three selected carbon sources were glucose, fructose and saccharose (as these are present in apple pomaces, which will be used as carbon sources in future studies), and the optimal relative proportions of those substrates were evaluated in Mixture Design experiments (Table 1).

**Table 1:** Composition of sugars in model mediums

Nr of medium	I	II	III	IV	V	VI	VII	VIII	IX	X
Carbon sources (g/L)										
Glucose	25	–	–	12.5	12.5	–	16.6	4.2	4.2	8.33
Frctose	–	25	–	12.5	–	12.5	4.2	16.6	4.2	8.33
Saccharose	–	–	25	–	12.5	12.5	4.2	4.2	16.6	8.34

## Materials and methods

### Inoculum preparation

The *P. freudenreichii* wild strain T82 from the collection of the Division of Biotechnology and Food Microbiology at Warsaw University of Life Science was used in the experiments.

The inoculum was prepared in a liquid VL (POCH) medium containing 2% glucose (pH 7.0). Inoculation cultures were carried out in 50 ml medium in Erlenmeyer flasks at 30 °C for 48 h. The inoculum for the production media was 10% of the media volume (v/v).

### Production media and growth conditions

The production medium included carbon sources (different proportions, Table 1), peptone (10 g/L), yeast extract (5 g/L), l-cysteine hydrochloride (0.4 g/L), potassium hydrogen phosphate (1.5 g/L) and dipotassium hydrogen phosphate (2.5 g/L). The initial acidity (pH) was 7.0. Cultures were carried out in 500 ml flasks containing 250 mL medium for 120 h at 30 °C, with 60 h under anaerobic conditions and 60 h under aerobic conditions (120 revolutions/min). The acidity of the medium during the fermentation process was adjusted every 12 h to pH 7.0 with 30% NaOH (acid neutralisation). The growth of bacteria in the medium was determined by measuring the optical density of the culture at λ = 550 nm (spectrophotometric method).

### Vitamin B12 analysis

Quantitative determination of intracellular vitamin B12 produced by *P. freudenreichii* T82 was performed using a microbial assay in amicroplate format, the VitaFast^®^Vitamin B12 assay (R-Biopharm). Vitamin B12 was extracted from biomass produced, acetate buffer and 1% NaCN were added to 1 g of biomass, the resulting mixture was shaken and subjected to a temperature of 95 °C, and finally the extract was diluted. In order to obtain the biomass yield, liquid culture was taken into previously dried and weighed centrifuge tubes. Samples were then centrifuged for 10 min (5600×g). The supernatant was decanted and the biomass obtained was dried at 70 °C for about 24 h to obtain a constant mass. Dried biomass was weighed and the result of the biomass yield was expressed as g/L. The medium and the diluted extract were added to the wells of the microplate coated with *Lactobacillus delbrueckii* subspecies *lactis* (*leishmanii*). The growth yield of *L. delbrueckii* depends on the amount of vitamin B12 in the culture environment as the bacteria grow until the entire vitamin B12 source is consumed. Samples were incubated in the dark at 37 °C for 48 h. Correlation of the growth of bacteria to levels of extracted vitamin B12 was measured in the form of turbidity (spectrophotometric measurement) and was read from a standard curve. Spectrophotometric measurements were made on a Bioscreen C microplate reader with a 540–650 nm filter.

### Determination of organic acids

Quantitative analysis of propionic and acetic acids obtained from fermentation was carried out using a gas chromatography with a flame ionization detector (GC-FID). Prior to the extraction of the acids, 25% sulfuric (VI) acid was added to the medium to release free organic acids from sodium propionate and sodium acetate (which resulting from alkalisation). The carboxylic acid fraction was extracted from the medium using a mixture of hexane (Avantor) and diethyl ether (Avantor) (1/1, v/v). The chromatographic separation was performed on a ZB-WAXplus column (30 m × 0.25 mm × 0.25 μm). Quantitative calculations were made with respect to an internal standard (undecanoic acid–C11:0, Sigma Aldrich) using correction factors. The acid quality analysis was performed based on a comparison of the retention times of the tested samples with the retention times of standards.

### Sugar analysis

The determination of the usage of reducing sugars by *P. freudenreichii* T82 bacteria was carried out using the 3.5-dinitrosalicylic acid (DNS) (the Miller method; Miller 1959). The principle of the method consists in the reducing effect of sugars present in the sample, which in an alkaline medium reduces the nitro groups of DNS to amino groups, the resulting amine derivatives are orange, and the intensity of the colouration is proportional to the sugar concentration of the tested sample. First, 1 mL of the solutions to be analysed was measured into test tubes, whilst 1 mL of distilled water was added to the control sample. Next, 1 mL of DNS and 5 mL of distilled water were added to all tubes and the contents of the tubes were mixed and placed in a boiling water bath for 5 min. After this time, the samples were cooled down and their absorbance was measured at λ = 550 nm. Saccharose containing samples were hydrolysed. The content of the reducing sugars in the analysed samples was read from a standard curve. To prepare the calibration curves, 8 solutions of glucose were prepared in the range 1–8 mg/mL (as described above).

### Statistical analysis

All experiments were performed in triplicate. The computer software Excel 2013 for Windows 10 and STATISTICA 10.0 for StatSoft. Inc. were used for mathematical and statistical calculations. Due to the need to perform multiple combinations of the three selected substrates, we used the mixture design method of DoE (Design of Experiment) as a tool to facilitate optimising the composition of carbon sources in the culture medium. The significance level was 0.05. In the experiments, simplex plans for the ternary mixtures were used.

## Results and discussion

In this study we used *P. freudenreichii* T82 strain for several reasons. Firstly, our preliminary studies have shown that this strain is the most promising for the production of propionic acid from pure sugars and apple pomaces, among all of others tested (Piwowarek et al. 2016). Furthermore, strains of *P. freudenreichii* have multiple enzymatic systems that allow them to use many carbon sources for metabolic processes (Deptula et al. 2017a). The literature also indicates that strains of *P. freudenreichii* are potentially more useful than the other bacteria of the genus *Propionibacterium* and relatives genus in the biosynthesis of propionic acid from pure carbon sources (sugars) and waste materials, and also have a metabolic system that makes possible to produce vitamin B12 (Yazdani and Gonzales 2007; Zhu et al. 2010; Khan et al. 2011; Chen et al. 2012; Deptula et al. 2017a). Moreover, *P. freudenreichii* strains have GRAS status granted by the US FDA (Food and Drug Administration), which allows the use of these bacterial cells and their metabolites in the production of food or animal feed. For example, *P. freudenreichii* is a dairy-associated bacterium, traditionally used in the production of Swiss type cheeses (Martens et al. 2002).

The growth of bacteria in a culture medium demonstrates the adaptation of microorganisms to specific conditions and their ability to use the nutrients provided, which serve the bacteria for a number of metabolic processes such as organic acid and vitamin B12 biosynthesis. For *P. freudenreichii* T82, a reduction of sugar content was observed in each of the tested media compared with the baseline (Table 2). The highest consumption of sugars (91%) by *P. freudenreichii* T82 was found in glucose exclusive medium (medium # I), which at the same time yielded the largest—among all variants—growth of bacteria (maximum OD value 2.8, Table 3). Increased consumption of carbon sources and substantial bacterial growth were observed in all types of media (media # IV, V and VII) where glucose amounted to at least half the available sugars. The greater the percentage of glucose, the higher the growth of the bacteria and the increased consumption of total sugars, which was presumably due to preferential glucose consumption by the tested *P. freudenreichii* strain. The lowest consumption of sugars was found in the medium with a 2.5% saccharose (27%, medium # III). The lowest growth (OD 1.4) was also observed in this medium (Table 3).

**Table 2:** Changes in the content of carbon sources in media during propagation of *Propionibacterium freudenreichii* T82

Carbon sources				Time (h)		Use (%)
Nr of medium	Glucose	Fructose	Saccharose	0	120	
	Sugars ratio in mediums			Carbon sources (g/L)		
I	1	0	0	25	2.3 ± 0.0	91
II	0	1	0	25	14.1 ± 0.2	43.7
III	0	0	1	25	18.2 ± 0.1	27.1
IV	0.5	0.5	0	25	7.6 ± 0.2	69.5
V	0.5	0	0.5	25	10.5 ± 0.2	57.9
VI	0	0.5	0.5	25	17.4 ± 0.3	30.4
VII	0.664	0.168	0.168	25	7.7 ± 0.1	69.3
VIII	0.168	0.664	0.168	25	16.1 ± 0.0	35.4
IX	0.168	0.168	0.664	25	16.3 ± 0.1	34.6
X	0.33	0.33	0.34	25	14.1 ± 1.0	43.7

During fermentation the pH was maintained at 7.0. Initial substrate concentration was 2.5%

**Table 3:** Changes in optical density (OD) in media during propagation of *Propionibacterium freudenreichii* T82

Nr	Time (h)				
	24	48	72	96	120
	OD				
I	1.4 ± 0.0	2.1 ± 0.0	2.4 ± 0.1	2.8 ± 0.1	2.8 ± 0.1
II	0.9 ± 0.0	1.2 ± 0.0	1.3 ± 0.0	1.5 ± 0.0	1.5 ± 0.0
III	0.8 ± 0.1	1.1 ± 0.1	1.3 ± 0.1	1.4 ± 0.0	1.4 ± 0.0
IV	1.4 ± 0.0	1.8 ± 0.0	2,0 ± 0.1	2.3 ± 0.2	2.1 ± 0.2
V	1.4 ± 0.1	1.9 ± 0.0	2.1 ± 0.1	2.2 ± 0.0	2.1 ± 0.1
VI	0.9 ± 0.0	1.1 ± 0.0	1.3 ± 0.0	1.5 ± 0.0	1.5 ± 0.0
VII	1.4 ± 0.0	1.6 ± 0.0	1.8 ± 0.0	2.1 ± 0.0	2.1 ± 0.0
VIII	1.2 ± 0.0	1.5 ± 0.0	1.9 ± 0.0	2.0 ± 0.0	2.0 ± 0.1
IX	0.8 ± 0.0	1.2 ± 0.0	1.5 ± 0.0	1.6 ± 0.0	1.5 ± 0.0
X	1.4 ± 0.1	1.6 ± 0.1	1.8 ± 0.0	1.9 ± 0.1	1.9 ± 0.0

During fermentation the pH was maintained at 7.0. Initial substrate concentration was 2.5%

Piwowarek et al. (2016) presented results that suggested the sugars that exert the most beneficial effects on the growth of the same strain of *P. freudenreichii* are fructose, glucose and saccharose. However, these cultures were conducted in media in which the optimum pH (7.0 for *Propionibacterium* species) was established only before inoculation of the production media in order to determine the growth and productivity of *P. freudenreichii* T82 in a stressful environment associated with increasing acidity. The biosynthesis of propionic and acetic acids by members of the genus *Propionibacterium* is inhibited by their negative feedback. Acidification of the medium by the release of metabolites (propionic and acetic acids) into the environment limits the growth of the bacteria and their metabolic activity. To minimise the effect of negative feedback by the acids synthesised, in this study the media were neutralised every 12 h with 30% NaOH. In addition to changing the sugar consumption profile, regular pH adjustment of the culture medium to 7.0 also improved the growth of the bacteria and significantly increased the production of propionic and acetic acids. Piwowarek et al. (2016) reported that the sum of these two acids produced in glucose containing medium was about 5 g/L, but with a regular alkalisation of the culture medium, the total acid content was here close to 53 g/L. In the 2016 study, the sugar that has the greatest influence on bacterial growth was fructose, compared with glucose in the present study. In addition to the carbon source, there are other factors that can significantly determine the result of the experiment and which are not included in the optimisation and modeling processes, such as the physiological state of bacterial cells in the inoculum or the environmental conditions of the cell culture, for example, pH. In both Piwowarek et al. (2016) and the present experiments, saccharose was the sugar that least supported bacterial growth. Most likely, this is because the hydrolysis of this disaccharide into simple sugars is related, among other things, to the production of suitable enzymes and increased energy input. Thus, it is easier for bacteria to use monosaccharides when available.

**Fig. 1:**
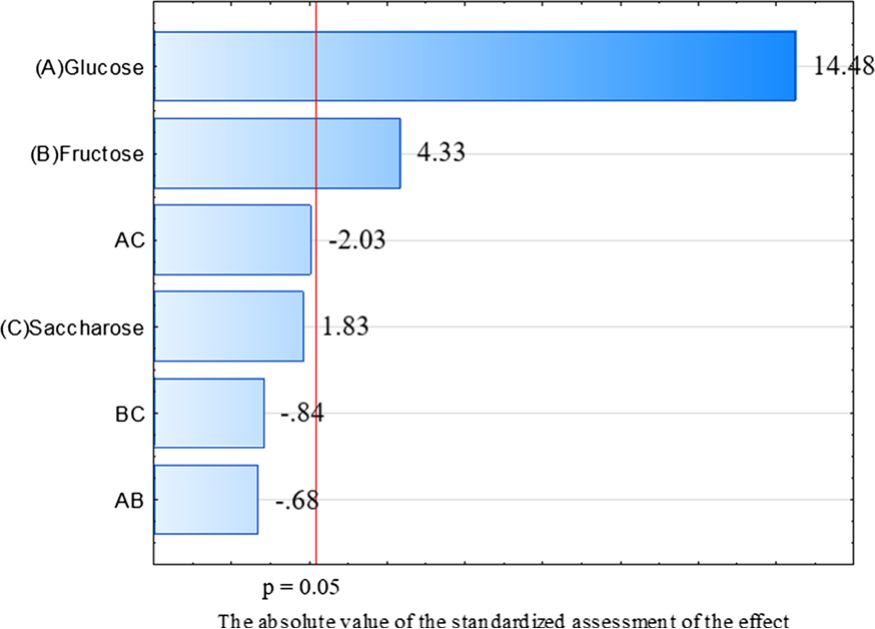
Pareto diagram showing the influence of the investigated factors on propionic acid production by *P. freudenreichii* T82 in 120 h of culture

**Fig. 2:**
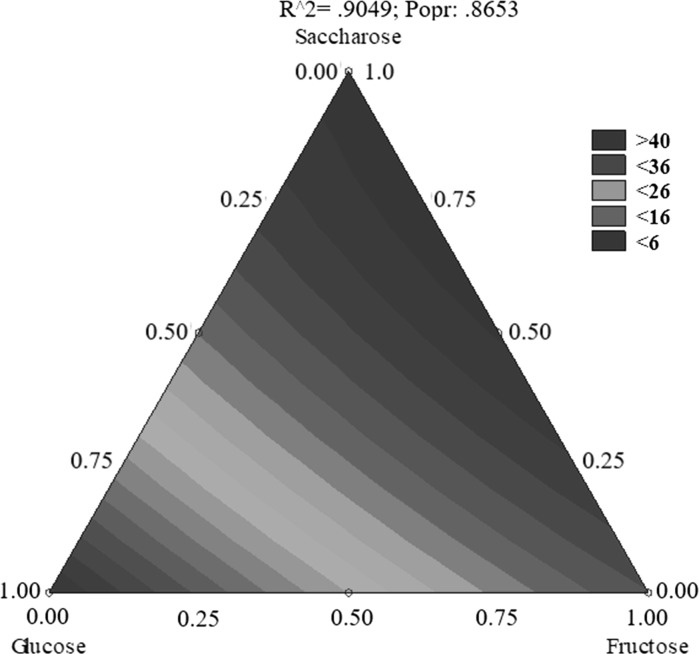
Contour plot demonstrating production of propionic acid by *P. freudenreichii* T82 depending on the composition of the carbon source in medium. The results obtained for medium I–X according to Table 5 were taken into account

Tables 4 and 5 show the course of propionic and acetic acids production by fermentation in the 10 tested media. The percentage of propionic and acetic acids varied during the fermentation process. The maximum production of the two metabolites by the tested bacterial strain, regardless of the culture conditions, was at 120 h. The highest concentration of produced acids was found in the media in which glucose accounted for at least 50% of the available carbon sources (# I, IV, and VII, Tables 3 and 4). *P. freudenreichii* strain T82 produced the smallest amount of acids in media # III and IV, that is, in the media in which the dominant carbon source was saccharose. Statistical analysis of the data fror propionic acid production by *P. freudenreichii* T82 showed that glucose (Figs. 1 and 2) was the sugar most important for the biosynthesis of this compound. Based on the results obtained, the use of sugar mixtures led to a reduction in acid production. The greater the percentage of glucose in the medium, the more efficient the biosynthesis of propionic and acetic acids. For the production of these metabolites by *P. freudnreichii* T82, glucose and fructose were found to be statistically significant. The statistical model obtained for the production of propionic acid by *P. freudenreichii* T82 strain was matched at 90% to the observed data (Fig. 2), so the model describes quite well the significant effect of carbon sources on the metabolic activity, and is also characterised by good prognostic abilities. This will enable the optimisation of the composition of carbon sources in media containing waste materials such as apple pomaces.

**Table 4:** Production of acetic acid by the *Propionibacterium freudenreichii* T82 strain

Nr	Time (h)				
	24	48	72	96	120
	Production of acetic acid (g/L)				
I	2.8 ± 3.0	5.1 ± 0.4	6.5 ± 1.4	11.4 ± 2.3	12.4± 0.6
II	0.5 ± 0.1	0.6 ± 0.3	1.5 ± 0.9	1.7 ± 0.2	3.2 ± 0.5
III	0.5 ± 0.0	0.6 ± 0.3	1.1 ± 0.5	1.2 ± 0.4	2.3 ± 1.8
IV	0.6 ± 0.3	3.1 ± 1.0	5.8 ± 2.1	3.7 ± 0.5	7.6 ± 0.8
V	0.5 ± 0.2	1.3 ± 0.3	1.6 ± 0.8	4.9 ± 0.7	8.2 ± 5.0
VI	0.6 ± 0.1	0.8 ± 0.2	1.1 ± 0.6	1.7 ± 0.0	2.8 ± 0.4
VII	0.6 ± 0.4	0.7 ± 0.7	1.2 ± 1.1	3.6 ± 1.1	10.6± 0.3
VIII	0.5 ± 0.3	0.5 ± 0.8	0.6 ± 0.0	1.4 ± 0.0	3.4 ± 0.4
IX	0.4 ± 0.1	0.4 ± 0.0	0.9 ± 0.0	2.5 ± 0.2	3.3 ± 0.8
X	0.5 ± 0.1	1.0 ± 0.2	1.9 ± 0.9	1.3 ± 0.4	6.2 ± 0.1

During fermentation the pH was maintained at 7.0. Initial substrate concentration was 2.5%

**Table 5:** Production of propionic acid by the *Propionibacterium freudenreichii* T82 strain

Nr	Time (h)				
	24	48	72	96	120
	Production of propionic acid (g/L)				
I	2.9 ± 1.6	5.9 ± 0.4	15.1 ± 6.1	29.0 ± 5.8	40.4 ± 1.2
II	0.6 ± 0.2	0.6 ± 0.3	2.0 ± 0.6	5.3 ± 1.4	12.1 ± 0.5
III	0.6 ± 0.4	0.7 ± 0.5	2.0 ± 0.7	3.7 ± 2.0	5.1 ± 2.8
IV	1.1 ± 0.5	2.4 ± 0.5	8.1 ± 2.8	15.4 ± 2.8	23.9 ± 1.1
V	1.2 ± 1.9	2.7 ± 1.3	6.8 ± 2.6	11.5 ± 2.3	15.8 ± 2.2
VI	0.6 ± 0.3	0.8 ± 0.2	1.9 ± 0.7	2.3 ± 0.2	5.7 ± 1.8
VII	0.4 ± 0.6	2.8 ± 1.1	4.0 ± 0.3	7.3 ± 2.4	23.2 ± 2.8
VIII	0.9 ± 0.1	2.9 ± 0.2	3.8 ± 1.2	5.4 ± 1.3	13.5 ± 2.0
IX	0.8 ± 0.1	1.6 ± 0.2	3.0 ± 0.3	4.9 ± 0.4	11.5 ± 0.3
X	1.3 ± 0.1	3.3 ± 0.5	5.8 ± 0.2	9.8 ± 0.7	16.2 ± 0.8

During fermentation the pH was maintained at 7.0. Initial substrate concentration was 2.5%

In this study, *P. freudenreichii* T82 strain has been shown to produce significant amounts of propionic acid from glucose as the sole carbon source. The theoretical yield of propionic acid production from glucose should be 0.55 g propionic acid/g substrate (Wang and Yang 2013). *P. freudenreichii* T82 strain could yield this metabolite at a rate of 1.6 g/g, almost 3 times higher than the theoretical yield and that (0.43 g/g) obtained by Wang and Yang (2013). The difference between the yields of propionic acid synthesis could be due to several reasons. One of them may be a naturally higher yield of propionic acid production by a particular bacterial strain. In addition, attention should be paid to differences in the applied culture parameters. Wang and Yang (2013) ran fermentations using *P. freudenreichii* subsp. *shermanii* DSM 4902 for 120 h at 32 °C and pH 6.5 (the pH was not stabilised during the process) and the initial glucose concentration was 3%. However, the present experiments used a temperature of 30 °C, a growth period of 120 h, a pH of 7.0 (pH neutralised every 12 h), and a carbon source concentration of 2.5%.

Optimal conditions for growth and metabolic activity of *Propionibacterium* species are typically 30 °C and pH 7.0. The use of other parameters may reduce the production of propionic acid. Failure to stabilise the pH in the Wang and Yang (2013) study likely caused accumulation of acids during the fermentation, which, by reducing the pH, had a negative impact on the growth and metabolism of the producer cells. Furthermore, a higher concentration of sugar (30 g/L), and therefore higher osmotic pressure of the culture medium, could adversely affect the cells.

Kośmider et al. (2010) used *P. freudenreichii* subsp. s*hermani* for fermentations conducted in media supplemented with glucose. Total sugar consumption occurred after 48 h. Synthesis of propionic acid reached 10.2 g/L with an efficiency of 0.5 g of propionic acid per gram of substrate. More propionic acid (15.7 g/L) was obtained by Zhu et al. (2010) after 120 h of fermentation. In comparison to the studies conducted by Kośmider et al. (2010) and Zhu et al. (2010) the amount of propionic acid obtained using *P. freudenreichii* T82 is high, confirming it. In contrast, Chen et al. (2012) obtained significantly higher concentrations of propionic acid (> 136 g/L) using immobilised *P. freudenreichii* strain CCTCC M207015 grown on sugar cane stalks.I Immobilisation also caused morphological changes in the bacterial cells including a threefold increase in length, decrease in diameter, and increase in surface area, which most likely allowed the more efficient absorption of nutrients from the media. Immobilisation may have also increased the resistance pf the strain to an acidified environment and limited the effect of negative feedback by propionic acid. Suwannakham and Yang (2005) came to similar conclusions. They immobilised a culture of *P. acidipropionici* ATCC 4875 inside a bioreactor, in order to reduce the effect of the acids on the metabolic activity of bacteria. Immobilised cells produced 20–59% more propionate (71.8 g/L), 17% less acetic acid and 50% less succinate compared to free cells.

Industrial production of vitamin B12 is based on fermentation processes using microorganisms, mainly genetically modified *P. denitrificans* (Blanche et al. 1995, 1998) which, due to the high efficiency of synthesis, accounts for 80% of global demand (Blanche et al. 1989; Miyano et al. 2000; Roman et al. 2001). The use of genetically modified microorganisms, however, raises a number of problems in social acceptance. For many years, various ways of efficient and industrially profitable production of vitamin B12 using non-genetically engineered microorganisms have been sought. Such research focuses on selection of suitable strains, carbon sources and culture methods (Quesada-Chanto et al. 1994; Gardner and Champagne 2005; Yu et al. 2015).

Among consumers, interest in food products enriched with therapeutic compounds such as vitamin B12 produced by in situ fermentation is growing. *P. freudenreichii* strains that produce therapeutically active vitamin B12 (with 5.6-dimethylbenzimidazole [DMBI] as the lower ligand in cobalamin) is of particular importance (Deptula et al. 2015, 2017b). Moreover, it has GRAS status, enabling the use of live cells in food and feed products.

**Fig. 3:**
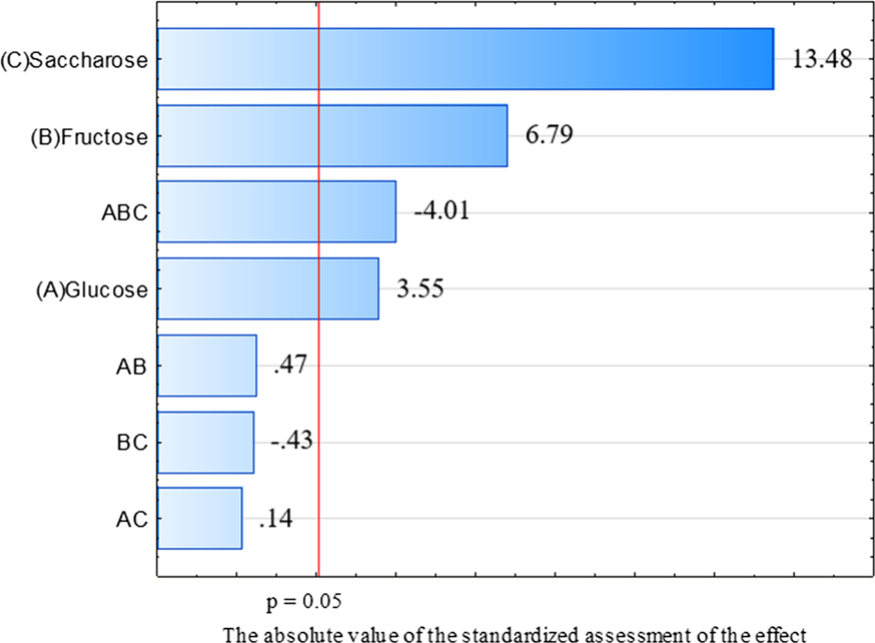
Pareto diagram showing the influence of the investigated factors on vitamin B12 production by *P. freudenreichii* T82 in 120 h of culture

**Fig. 4:**
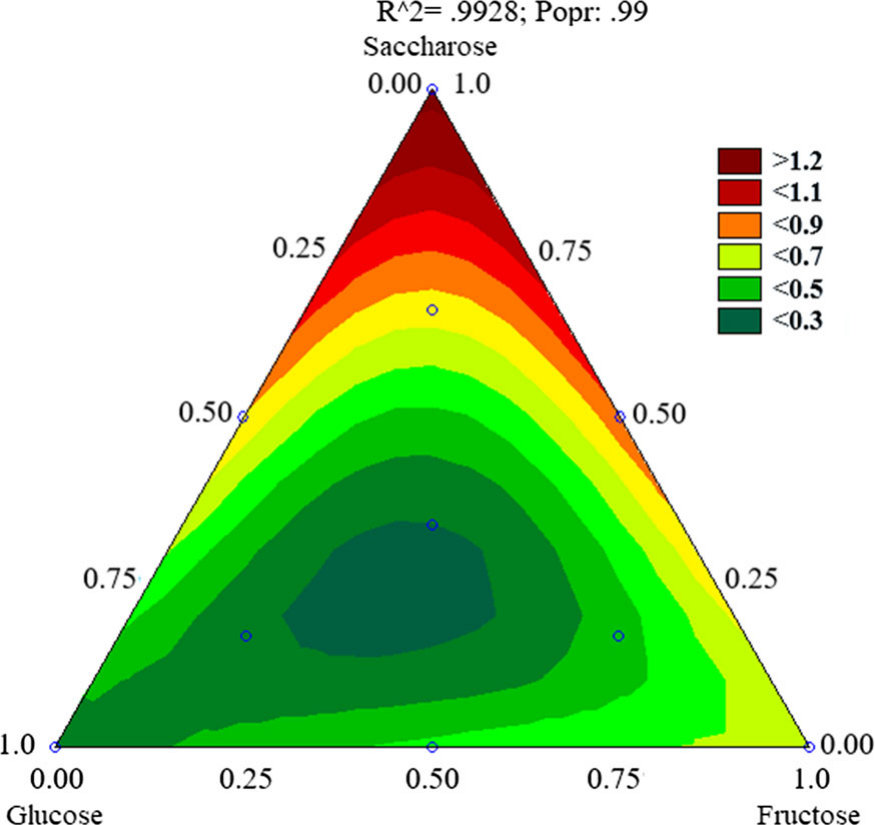
Contour plot demonstrating production of vitamin B12 by *P. freudenreichii* T82 depending on the composition of the carbon source in medium. The results obtained for medium I–X according to Table 6 were taken into account

In case of the production of vitamin B12 by *P. freudenreichii* T82, the results were quite different (Table 6). In the media where the growth of bacteria and acid production were the least, that is, in media III and V, the highest production of vitamin B12 was found, close to 1.4 μg/100 mL. Conversely, in media where growth and acid production was the highest (media # I and IV), the lowest production of cobalamin was found, ~ 0.3 μg/100 mL (Table 6). This is most probably because the optimum pH for the production of vitamin B12 by the *P. freudenriechia* T82 strain is 7.0. Although the pH of the media was neutralised every 12 h, there was a sharp decrease in the pH between the neutralising steps, especially in the media where acid biosynthesis was most notable (medium # IV), and thus there was disturbance of optimum conditions for cobalamin biosynthesis by *P. freudenreichii* T82. Vitamin B12 is used by propionic acid bacteria as a cofactor of the enzymatic reaction for the production of propionic acid. Coenzyme B12 and methylmalonyl CoA mutase are responsible for converting succinyl-CoA into methylmalonyl-CoA, which is the penultimate intermediate in the propionate biosynthesis pathway. With the increased production of propionic acid (media # I and IV), the consumption of cobalamin increases for the aforementioned reaction, which in turn leads to a decrease in the vitamin B12 yield. The statistical analysis of the results of vitamin B12 production showed that the sugar most important for the biosynthesis of this compound by *P. freudenreichii* T82 was saccharose (Figs. 3 and 4). The addition of glucose and/or fructose resulted in reduced metabolite production. In the medium in which saccharose formed at least 50% of sugars, increased production of cobalamin, reduced acid production, and reduced sugar consumption compared with the other media was observed. The increased synthesis of cobalamin in these media probably reflects the pH of the medium (close to 7.0, the optimum value for cobalamin biosynthesis) caused by the low content of propionic and acetic acids. In addition, because of the small amount of saccharose used for acid synthesis (preferential use of glucose), saccharose could be used to produce cobalamin. It may be that the use of saccharose as a carbon source limits the production of acids and stimulates the biosynthesis of vitamins at the same time.

**Table 6:** Production of vitamin B12 by the *Propionibacterium freudenreichii* T82 strain

Carbon sources				Time (h)
	Glucose	Fructose	Saccharose	120
Nr	Sugars ratio in mediums			Vitamin B12 (µg/100 mL)
I	1	0	0	0.32 ± 0.00
II	0	1	0	0.54 ± 0.02
III	0	0	1	1.33 ± 0.03
IV	0.5	0.5	0	0.43 ± 0.03
V	0.5	0	0.5	0.88 ± 0.04
VI	0	0.5	0.5	1.39 ± 0.04
VII	0.664	0.168	0.168	0.35 ± 0.00
VIII	0.168	0.664	0.168	0.71 ± 0.02
IX	0.168	0.168	0.664	0.43 ± 0.01
X	0.33	0.33	0.34	0.31 ± 0.01

During fermentation the pH was maintained at 7.0. Initial substrate concentration was 2.5%

The amount of vitamin B12 obtained from *P. freudenreichii* strain T82, irrespective of the media used, did not indicate a natural ability to produce cobalamin with high efficiency, even though the pH was adjusted every 12 h and the temperature of the culture was maintained at an optimum level. Here the production media were not supplemented with compounds important for the production of cobalamin (such as cobalt ions, DMBI, glycine, threonine, 5-aminolevulinic, or choline), in order to determine the natural ability for synthesis of this vitamin by *P. freudenreichii* T82. Therefore it seems, in order to improve the efficiency of vitamin B12 biosynthesis by this strain, production media should be enriched in cobalamin biosynthesis precursors to direct metabolism towards the production of this specific metabolite (Roman et al. 2001; Murooka et al. 2005; Thirupathaiah et al. 2012; Kośmider et al. 2012; Wang et al. 2015; Deptula et al. 2017b). Chamlagain et al. (2016) investigated the effect of addition of DMBI precursors (riboflavin [RF] and nicotinamide [NAM]) and DMBI alone on the production of vitamin B12 by *P. freudenreichii*. Supplementation of media with riboflavin (40 mM) and nicotinamide (27 mM) increased the yield of cobalamin fourfold as compared with that in control cultures, as did supplementation with DMBI (100 mM).

To increase the production of vitamin B12 by *P. freudenreichii* T82 strain, it is likely necessary to inhibit acid biosynthesis or to remove them from the culture medium, for example, by fermentation combined with purification in an activated carbon absorption column, extractive fermentation, electrodialysis, or the use of immobilised cells. Growth of *P. freudenreichii* T82 in larger volumes of production medium in an appropriately controlled, bioreactor environment could also improve the productivity of cobalamin biosynthesis. Wang et al. (2015) examined the effect of propionic acid and DMBI on vitamin B12 biosynthesis by *P. freudenreichii* during fermentation combined with purification in an absorption column in a bioreactor medium. They found that maintaining a concentration of propionic acid at the initial stage of fermentation at 10–20 and 20–30 g/L at a later stage can effectively increase cobalamin production, to 58.8 mg/L. Wang et al. (2012) examined an expanded bed adsorption bioreactor for the simultaneous biosynthesis of propionic acid and vitamin B12 by *P. freudenreichii* CICC 10019. They succeeded in recovering propionic acid by semi-continuous recirculation of the unfiltered broth, eliminating the feedback effect of this metabolite, which resulted in concentrations of propionic acid and vitamin B12 of 52.5 g/L and 43.04 mg/L, respectively. Vitamin B12 production by *P. freudenreichii* T82 could also be improved by using more suitable carbon sources. Wang et al. (2014) were able to produce high yields of vitamin B12 (0.72 mg/g) and propionic acid (0.81 g/g) in *P. freudenreichii* subsp. *shermanii* using concurrent glucose and glycerol fermentation.

One of the major problems of most industrial plants is finding uses for the by-products of technological processes. Therefore, innovative waste management solutions such as biotechnology are being sought. The biotechnological use of bacteria may reduce the pollution of the environment not only by waste disposal, but also by converting it into useful and valuable industrial ingredients. The high yield of propionic acid biosynthesis by *P. freudenreichii* T82 provides an approach for the use of this strain to naturally obtain this compound, which is currently produced mainly by expensive and environmentally harmful petrochemical processes (e.g. hydrocarboxylation of ethylene). The cost of acquiring one ton of propionic acid via biotechnological process using microorganisms reaches up to US $2000, which is 2 times the cost of chemical acid production. However, due to scarcity of resources, serious environmental damage resulting from the current production and a growing demand for natural and organic food, there is a growing interest in obtaining propionic acid using microorganisms and using waste materials from various industries as media (which should reduce the cost of acid production) (Baumann and Westermann 2016). In order to make the tested strain of *P. freudenreichii* T82 useful for industrial production, further research is needed on the selection of a suitable carbon source in the form of waste materials that contain significant quantities of glucose and fructose (as in apple pomaces) and to determine culture methods which allow for increased production of acid (e.g. immobilisation or growth in a bioreactor). *P. freudenreichii* T82 can also produce cobalamin, but the efficiency of this process is lower. Further studies on the biosynthesis of this metabolite by the *P. freudenreichii* T82 strain are needed, such as the optimisation of culture conditions (e.g. suitable carbon sources, biostimulators), maintaining cultured cells, or the regulation of biosynthesis of organic acids resulting from the fermentation, for example, by using carbon source limiting their production and stimulating the vitamin biosynthesis. Statistical analysis (DoE) of the results obtained allowed us to determine which sugars (carbon sources) exert the most beneficial influence on the biosynthesis of propionic acid and cobalamin. This will allow for the selection of appropriate quantities of apple pomaces and, as a result, the culture medium based on these waste materials will contain optimum concentrations of suitable sugars, thus improving the efficiency of propionic acid and vitamin B12 production by *P. freudenreichii* T82.

The datasets during and/or analysed during the current study are available from the corresponding author on reasonable request.
